# Correction: The emotion regulation effect of cognitive control is related to depressive state through the mediation of rumination: An ERP study

**DOI:** 10.1371/journal.pone.0228807

**Published:** 2020-01-31

**Authors:** The PLOS ONE staff

The images for Figs 1, 2 and 3 are incorrectly switched. The image of Fig 1 should appear as Fig 3, the image of Fig 2 should appear as Fig 1, and the image of Fig 3 should appear as Fig 2. The figure captions appear in the correct order. The publisher apologizes for the errors.

**Fig 1:**
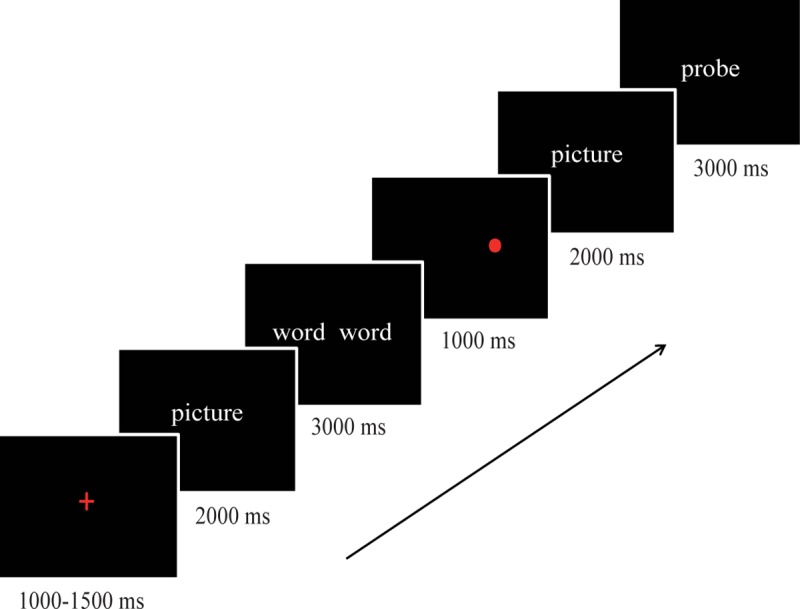
The display sequence and timing in the trials.

**Fig 2:**
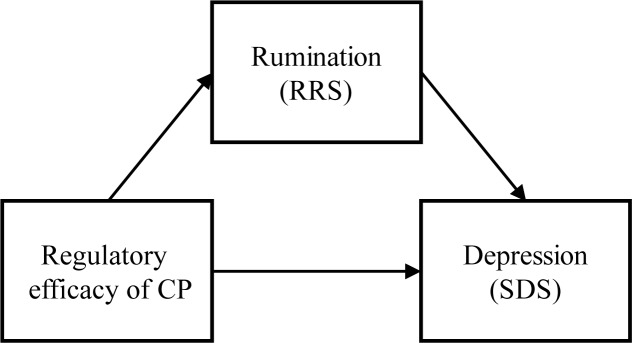
Model of mediation analyses.

**Fig 3:**
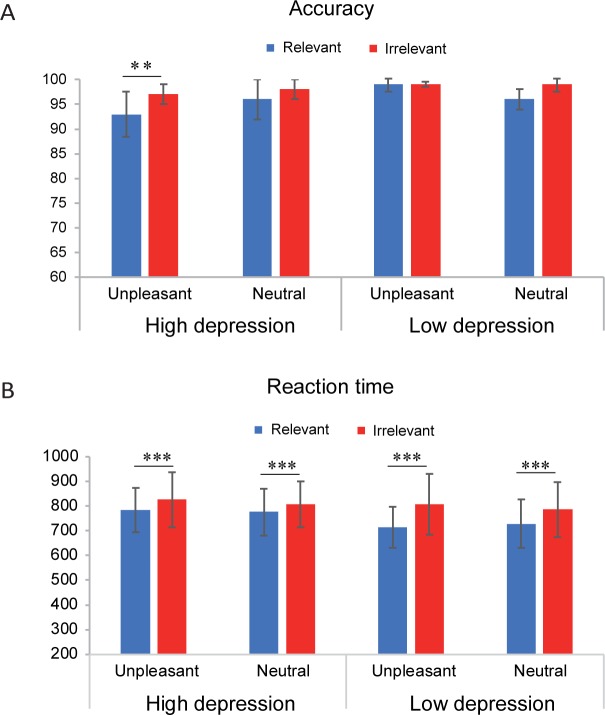
Mean accuracy (A) and reaction time (B) of recognition for the probe words in different conditions. Error bars indicate standard deviations. ** p < 0.01, *** p < 0.001.
