# Supplementary material for: The emotion regulation effect of cognitive control is related to depressive state through the mediation of rumination: An ERP study
Source: PLoS One. 2019 Nov 15;14(11):e0225285. doi: 10.1371/journal.pone.0225285 (PMC6857946; doi:10.1371/journal.pone.0225285)
Supplement: Attachment — Submitted filename: review comments.docx [file pone.0225285.s004.docx]

The current study examines emotion regulation effect in participants with low and high depression level. The authors analyze behavioral and ERP data. They concluded that the underlying mechanisms of the link between the function of cognitive control in emotion generation and depressive symptoms, and indicated the pathway from the regulatory efficacy of cognitive control to depression via rumination.

The research question is interesting and has the potential to increase our understanding of emotion regulation in depression.

However, I have major concerns and I think that the data is not adequately analysed and interpreted and I cannot recommend publishing the paper in its current form.

 I will outline my main concerns and comments below:

1. The title used ‘depressive symptom’. However, the participants in the current study were ones with low and high depressive level. Maybe the ‘depressive sate’ was proper.

2. The theoretical basis of this paper is based on patients with depression. However, ordinary college students with high SDS scores selected in this experiment can only be regarded as trait population, so whether the theoretical basis is universal in the study of trait population. Relevant literature is not listed for illustration.

3. The data analysis section, the authors use the regression methods to remove Vertical ocular artifacts. In fact, the ICA method was more reliable than regression procedure for artifacts removing. Why the authors don’t use the ICA method?

4. Previous studies have confirmed that theta-band oscillations are critical for cognitive control. I suggest that authors doing time-frequency analysis to explore the TF information in the data.

5. In the results section, what’s the mean of ‘total’ in the table 4. It was not correct to calculate the regression between all participants and LPP amplitudes, as all participants were not continuous variable.

6. The figure was not marked clearly. What’s the figure in three rows represent in Fig4 and Fig5? The statistical value should be written in Fig6.

7. In Table3, the early LPP amplitude in the high depression group and the low depression group showed significant differences under the conditions of cp-u and NEU. However, in the middle and late windows, the differences in the high depression group under the conditions of cp-u and NEU gradually disappeared, and the differences in the low depression group varied, which was not explained in the discussion.

8. The written English needs considerable improvement.
