# Supplementary material for: The emotion regulation effect of cognitive control is related to depressive state through the mediation of rumination: An ERP study
Source: PLoS One. 2019 Nov 15;14(11):e0225285. doi: 10.1371/journal.pone.0225285 (PMC6857946; doi:10.1371/journal.pone.0225285)
Supplement: Attachment — Submitted filename: Response to reviewers.docx [file pone.0225285.s005.docx]

**Response to reviewers**

Dear editor:

We are truly grateful to your and reviewers’ comments and thoughtful suggestions on our manuscript (PONE-D-19-20025). Based on the comments we received, careful modifications have been made to the manuscript. Changes to our manuscript were all highlighted in the version with track-changes. Point-by-point responses to reviewers’ comments are listed below. We thank you and the reviewers for the time and efforts on our paper. We look forward to hearing from you.

Wish you all the best!

Sincerely yours,

Shuzhen Gan

**Reviewer #1:**

**1. The title of the paper will cause ambiguity, from which the research variables and topics cannot be clearly understood. The match between the topic and the key words is not high.**

**Response:** Thanks for the constructive advice and we apologize for the ambiguity of the title. The main findings of our study are the emotion regulation effect of cognitive control varies with depression level, and rumination serves as a mediator in the relationship between the emotion regulation effect of cognitive control and depression. Therefore, we have changed the title to “The emotion regulation effect of cognitive control is related to depressive state through the mediation of rumination” in the revised manuscript. We feel that this title might be clearer and more appropriate for our study. As our study focused on a specific type of cognitive control—controlled processing different semantic representations, we added the “semantic representation” as a key word, and change “emotion” to “emotion regulation”. Thus, the key words in our paper have been modified to “cognitive control, depression, emotion regulation, semantic representation, rumination, late positive potential”. We feel that these key words might be more match our research topic.

**2. In participants section, ” Participants were paid 20 yuan after completing the SDS questionnaire and 80 yuan after the ERP experiment”. But there's nothing about the RRS for testing. When to test? How about payment?**

**Response:** Thank you very much for pointing out this problem and we regret for this negligence. The rumination tendency of all participants was tested using RRS immediately after the ERP experiment. And the payment for completing RRS and EEG experiment together was 80 yuan. In the revised manuscript, we have added these descriptions in page 9 line 182-183, and page 14, line 306-307 in the revised manuscript with track changes.

**3. Why not to use Emotion Image Library in Chinese Version?**

**Response:** Thank you very much for pointing out this problem. Before selecting the experimental materials to evoke participants’ emotion, we researched literature in the field of emotion and emotion regulation, and found that the vast majority of studies use pictures in International Affective Picture System (IAPS) to evoke participants’ emotional response including the emotion-enhanced ERPs such as LPP. The IAPS is a standardized tool which is widely used to induce emotions. At the same time, we also considered the potential culture difference on its use in Chinese population. We found that IAPS has been demonstrated to have satisfactory reliability and validity on emotion-elicitation in Chinese samples. The ratings on valence and arousal in Chinese old adults have high internal consistency and low inter-individual variance, and the ratings also have high similarities and small difference between Chinese and German older adults (Gong, Wang, 2016). In addition, the emotionally arousing pictures in IAPS elicit larger amplitude of LPP than neutral ones and the amplitude is related with self-rating measures in Chinese participants (Yen, et al., 2010). It indicated that the IAPS pictures induced emotion-related subjective feelings and psychophysiological responses are universal rather than culture-specific.

To confirm IAPS pictures used in our experiment can elicit emotional response in Chinese college students as in those Caucasian population, we recruited 50 participants whose age and education level are similar with those participated in the ERP experiment. The results were consistent with those found in Lang’s study (2005), revealing that the aversive IAPS pictures rated as more unpleasant and higher arousal than the neutral ones. These results are described in page 10-11, line 218-229 in the version with track changes.

For above reasons, we used IAPS pictures as emotion-eliciting pictures in order to increase the comparability of our results with the existing studies in the research field of emotion and emotion regulation.

**Reviewer #2:**

**1. The title used ‘depressive symptom’. However, the participants in the current study were ones with low and high depressive level. Maybe the ‘depressive sate’ was proper.**

**Response:** Thank you very much for the suggestion. We agree that the ‘depressive state’ is a more proper term in the title. In the revised manuscript, we have replaced the ‘depressive symptom’ with “depressive state” in the title (page 1, line 1-3 in the revised manuscript with track changes).

**2. The theoretical basis of this paper is based on patients with depression. However, ordinary college students with high SDS scores selected in this experiment can only be regarded as trait population, so whether the theoretical basis is universal in the study of trait population. Relevant literature is not listed for illustration.**

**Response:** Thank you very much for pointing out the lack of literature from nonclinical population and we regret for this fault.

We have researched the literature, and found that the nonclinical individuals with high depression level also have emotion dysregulation as those clinical ones; they have decreased use of adaptive regulatory strategies such as reappraisal in which cognitive control is involved, and increased use of maladaptive strategies such as rumination (Garnefski, Kraaij, 2006). High depression level is also associated with various cognitive deficits including attention disengagement, inhibition, interference control, etc. (Derakshan, et al., 2009; Koster, et al., 2005; Palacio-Gonzalez, et al., 2017). With the possible role of cognitive control in emotion regulation, these studies supported the hypothesis that the deficits of cognitive control might contribute to the emotion regulation failure and increase the depression level in nonclinical samples. The mediation role of rumination in the relationship between the abilities of cognitive control and depressive symptoms in nonclinical populations has also been found (DeJong, et al., 2019) (page 6, line 124-125 in revised manuscript with track changes), which support the hypothesis that rumination might mediate the relationship between the emotion regulation effect of cognitive control and depressive symptoms.

In the revised manuscript, we have described relevant findings in nonclinical samples in the Introduction in page 5 line 96-101, line 106-108, and page 6, line 120-122, line 124-125. Relevant references have also been listed in Reference section. We feel that the inclusion of these findings has strengthened the theoretical the basis of our study.

**3. The data analysis section, the authors use the regression methods to remove Vertical ocular artifacts. In fact, the ICA method was more reliable than regression procedure for artifacts removing. Why the authors don’t use the ICA method?**

**Response:** Thank you very much for pointing out this problem.

In the manuscript, we preprocessed the EEG data with Neuroscan 4.5 software in which the regression procedure devised by Semlitsch and his colleagues is used to remove the vertical ocular artifacts (Semlitsch et al., 1986). It is a widely used correction procedure in those EEG data collected by Neuroscan system. And the reliability and validity has also been demonstrated.

As one of the various regression methods for removing vertical ocular artifacts, Semlitsch and his colleagues have demonstrated the advantages of their method (Semlitsch et al., 1986). First, it can remain trials for further analysis as much as possible. Secondly, this correction method reduces coherence between VEOG artifacts and ongoing EEG by using all eye-blinks as trigger to average VEOG and EEG, thereby all blinks in the experiment were involved in the regression analysis. This leads to a high reliability and validity of regression factors, and helps to remove vertical ocular artifacts as much as possible and obtain clear EEG data.

We agree that ICA method is also a widely-used method to correct vertical ocular artifacts for its good reliability. However, we feel that we might have two limitations to use ICA method in our data. Firstly, ICA method requires the users to manually select EOG components for correction, it might lead to analyzing bias caused by subjective experiences, and it also creates challenges for implementing automated correction routines. Secondly, as we concern about the frontal ERP, we are not sure whether the removing of ocular artifacts with ICA will also remove the EEG signals in frontal electrodes.

To investigate whether our results will be influenced by these two different correction methods, we re-analyzed the EEG data with EEGLAB (Delorme and Makeig, 2004), an open-source toolbox running in the MATLAB environment. All the preprocessing steps were similar except the use of ICA method to remove vertical ocular artifacts. The grand-average waveforms for the pictures presented for the first time in the trials were shown in **SFig 1**. The emotion effect in the high and low depression groups were tested with a group (high, low) × emotion (aversive, neutral) × site (anterior, posterior) ANOVA in 500-1000, 1000-1500, and1500-2000 ms, respectively.

The results indicated a significant emotion effect on LPP amplitude in all three time windows, with aversive pictures evoking larger amplitudes than neutral pictures (early: *F*(1,47) = 42.14, *p* < 0.001, *η_p_^2^* = 0.47; middle: *F*(1,47) = 29.71, *p* < 0.001, *η_p_^2^* = 0.39; late: *F*(1,47) = 8.14, *p* < 0.01, *η_p_^2^* = 0.15). The picture-evoked LPPs in anterior electrodes and posterior electrodes were different in all three windows (early: *F*(1,47) = 13.51, *p* < 0.01, *η_p_^2^* = 0.22; middle: *F*(1,47) = 5.63, *p* < 0.05, *η_p_^2^* = 0.11; late: *F*(1,47) = 8.56, *p* < 0.01, *η_p_^2^* = 0.15). In addition, the interaction between the emotion and site in the early window was found (*F*(1,47) = 15.47, *p* < 0.001, *η_p_^2^* = 0.24). Further simple effect analysis indicated that in both of anterior and posterior sites, aversive pictures evoked larger LPPs than neutral pictures (anterior: *F*(1,47) = 22.97, *p* < 0.0001, *η_p_^2^* = 0.33; posterior: *F*(1,47) = 59.41, *p* < 0.0001, *η_p_^2^* = 0.56). These results suggested that the emotion effect during the presentation of the first picture using ICA method were consistent with that using regression procedure by Semlitsch’s team.

**SFig 1. Grand average waveforms for the pictures presented for the first time in the high (left) and low (right) depression groups, respectively.**

The grand-average waveforms for the pictures presented for the second time in the trials were shown in **SFig 2**. The effect of controlled-processing on LPP in the high and low depression groups were tested with a group (high, low) × CP (CP-U, CP-N, NEU) × site (anterior, posterior) ANOVA in 500-1000, 1000-1500, and 1500-2000 ms, respectively.

The results indicated that the CP effect is significant in the first and second windows (early: *F*(1,47) = 34.03, *p* < 0.001, *η_p_^2^* = 0.42; middle: *F*(1,47) = 12.55, *p* < 0.001, *η_p_^2^* = 0.21) and the site effect is significant in the early window (*F*(1,47) = 5.00, *p* < 0.05, *η_p_^2^* = 0.10). The CP effect in the late window and the site effect in the middle window that were significant in previous results with regression procedure were not observed.

The results of planned multiple comparisons were shown in STable 1. Bonforroni Correction was applied. Some different results were found compared with the previous results using regression method, and these results were highlighted with red color. For instance, CP-U enhanced the LPP amplitudes than NEU with a longer time (from 500-1000 ms to 500-1500 ms) in parietal site but with a shorter time (from 500-1500 ms to 500-1000 ms) in anterior site in high depression group, with a longer time (from 500-1500 ms to 500-2000 ms) in parietal site in low depression group. And CP-N elicited larger LPP amplitudes than NEU in the early and middle windows in anterior site in the low depression group, and had no difference with NEU in the early window in anterior sites in the high depression group.

However, the main results here were consistent with those using regression method. Specifically, CP-U elicited significantly larger LPP than NEU in both high and low depression groups which indicate an emotion effect. And this effect is gradually disappeared in the middle and late windows in the high depression group. CP-N decreased LPP amplitudes compared with CP-U in the parietal electrodes in the early window in the low depression group, which suggested a regulatory effect of controlled-processing different meanings. And this regulatory effect was not found in the high depression group.

In all, although there were a few differences between the ERP results using ICA method and the ERP results using regression method, the main results were replicated in the analysis using ICA method. It demonstrated the reliability of our results.

**SFig 2. Grand average waveforms for the pictures presented for the second time in the high (left) and low (right) depression groups, respectively.**

**STable 1. Results of t-tests among CP-N, CP-U and NEU conditions.**

|  | **500-1000** | | | **1000-1500** | | | **1500-2000** | | |
| --- | --- | --- | --- | --- | --- | --- | --- | --- | --- |
|  | *df* | *t* | *d* | *df* | *t* | *d* | *df* | *t* | ***d*** |
| **High depression** |  |  |  |  |  |  |  |  |  |
| anterior CP-N vs. CP-U | 25 | 0.90 | — | 25 | 1.05 | — | 25 | 0.69 | — |
| anterior CP-U vs. NEU | 25 | 3.89** | 0.76 | 25 | **2.47** | — | 25 | 1.62 | — |
| anterior CP-N vs. NEU | 25 | **2.62** | — | 25 | 1.2 | — | 25 | 1.01 | — |
| parietal CP-U vs. CP-N | 25 | 0.01 | — | 25 | -0.1 | — | 25 | -0.8 | — |
| parietal CP-U vs. NEU | 25 | 6.10*** | 1.2 | 25 | **2.97*** | 0.58 | 25 | 0.83 | — |
| parietal CP-N vs. NEU | 25 | 5.17*** | 1.01 | 25 | 1.69 | — | 25 | 1.06 | — |
| **Low depression** |  |  |  |  |  |  |  |  |  |
| anterior CP-U vs. CP-N | 22 | 2.56 | — | 22 | 1.48 | — | 22 | 1.29 | — |
| anterior CP-U vs. NEU | 22 | 4.94*** | 1.03 | 22 | 4.81*** | 1 | 22 | 3.60* | 0.75 |
| anterior CP-N vs. NEU | 22 | **3.2*** | 0.67 | 22 | **3.15*** | 0.67 | 22 | 1.98 | — |
| parietal CP-U vs. CP-N | 22 | 3.62* | 0.76 | 22 | 1.48 | — | 22 | 1.59 | — |
| parietal CP-U vs. NEU | 22 | 5.48*** | 1.14 | 22 | 4.60*** | 0.95 | 22 | **3.68**** | 0.77 |
| parietal CP-N vs. NEU | 22 | 3.21* | 0.67 | 22 | 3.64** | 0.76 | 22 | 2.29 | — |

**4. Previous studies have confirmed that theta-band oscillations are critical for cognitive control. I suggest that authors doing time-frequency analysis to explore the TF information in the data.**

**Response:** Thank you very much for the constructive advice. We agree that the time-frequency analysis will help to provide more evidences for the cognitive control in our experimental task.

After the cue dot presented, one of the two words was perceived as target word, the other one was perceived as to be irrelevant word, instructed by the location the cue dot. We hypothesized that under the CP-N condition, participants had to resolve the interference from the irrelevant unpleasant words and executive their attention to the goal-relevant neutral words relative to that under the CP-U and NEU conditions. Therefore, these differences were investigated in the time-frequency analysis.

In the time-frequency analysis, the raw data were preprocessed in the EEGLAB as below. Continuous data was re-referenced to the average of left and right mastoids. Then a bandpass of 0.1-30 Hz was used to filter the data. Vertical ocular artifacts were removed by using ICA method. Then the data were segmented to epochs ranging from the -500 ms before the cue dot onset to 1000 ms after the cue dot onset. However, as we didn’t send a mark when the cue dot onset, we had to segment the EEG data into epochs according to the mark of the picture presented for the first time in each trial (4000 ms before the cue dot). Thus, 3500 ms to 5000 ms after picture onset was epoched. As no event mark exists in this duration, we cannot categorize trials into their corresponding conditions according to the mark type which indicating different conditions. Instead, we had to categorized these trials into the corresponding conditions according to their presented sequence in the experiment. This introduced a big loss of data. The total number of trials of the experiment is 135, 45 for each condition, CP-U, CP-N, and NEU. However, due to recording error, several trials lost for some participants. This make it difficult to correspond each trial with condition correctly according to the presented sequence in the experiment. Finally, only 34 participants (17 in high depression group and 17 in low depression group) with full number of trials entered for further time-frequency analysis. A Fast Fourier with fixed 400 sliding Hanning window. The spectrogram was baseline-corrected (reference interval: from 3600 to 3900 ms after picture onset, i.e., -400 to -100 ms before cue dot onset) by subtracting the signal power of each frequency averaged within the reference interval from the signal power at each timepoint in each frequency.

Based on the visual inspection on the time-frequency data (see SFig 3), we found that the oscillations in delta-theta range responded to the cue dot in the window 300-450 ms after cue dot onset (4300-4450 ms after picture onset as displayed in SFig 3). And previous studies also found that mid-frontal oscillations in the delta-theta band (2-7 Hz) have been related with cognitive control (Kaiser, Schutz-Bosbach, 2019). Therefore, we calculated the power in the frequency range of 2-7 Hz in the time window of 300-450 ms after cue dot onset.

A group (high, low depression)×CP (CP-N, CP-U, NEU) ANOVA was conducted on the power of the delta-theta oscillations. The CP effect was marginally significant (*F*(*2,64*) = 2.97, *p* = 0.06, *η_p_^2^* =0.09), with CP-N elicited significantly larger delta-theta power than CP-U (*t*(*33*) = 2.25, *p* < 0.05). The comparisons between the CP-N and NEU, the CP-U and NEU were not significant (*ps* > 0.05). In addition, the group effect was not significant (*F*(*1,32*) = 1.89, *p* = 0.18). And the interaction between the group and CP was not found (*F*(*2, 64*) = 0.70, *p* = 0.50). These results were partly consistent with previous studies. Larger delta-theta power in CP-N reflected the cognitive control efforts to inhibit the interference from irrelevant negative information (Kaiser, Schutz-Bosbach, 2019). In addition, although the group effect was not significant, we observed a tendency of larger delta-theta power in high depression group than that in low depression group. This result is consistent with previous studies found in clinical depressed patients and depression-prone individuals, and might reflect their general cognitive slowing and increased effort to complete cognitive tasks compared with healthy controls and low depression individuals (Knyazev, et al., 2016).

**SFig 3. Time-Frequency maps for the cue dot in midfrontal electrodes (FCZ) in the high and low depression groups.**

The results of time-frequency analysis provided evidences for cognitive control involved in our experimental task and implied the potential group difference. However, these data were not included in the revised manuscript for several reasons. Firstly, only EEG data from 34 participants were included in the time-frequency analysis, which due to no marks were sent at the onset of cue dot. Secondly, the results of time-frequency analysis were possibly contaminated by signals of previous words. We didn’t set an interval between the words-presentation and cue dot-presentation in experimental design. The -500 ms baseline used for the analysis of 1000 ms cue dot is the period of the presentation of previous words. Moreover, the previous words were different types in different trials, such as unpleasant-neutral pair (for aversive picture) and neutral-neutral pair (for neutral picture), which also will influence the time-frequency analysis of later-presented cue dot.

**5. In the results section, what’s the mean of ‘total’ in the table 4. It was not correct to calculate the regression between all participants and LPP amplitudes, as all participants were not continuous variable.**

**Response:** Thank you very much for pointing out this important problem. The “total” in Table 4 means all participants including the high and low depression groups. We regret for the improper description on the participants recruitment for the ERP experiment, which suggested the SDS scores of the two groups were not continuous. In fact, after the volunteers completing the SDS, we selected a cutoff score of 50, scores below which indicating normal population without depressive symptoms, scores equal to and above which indicating mild to severe depressive symptoms (Zung, 1972). Then we try to selected the students with highest scores above the cutoff score 50 into high depression group, and the students with lowest scores below the cutoff score 50 into low depression group. However, only part of the students volunteered to take part in the following ERP experiment. Therefore, the participants with the high and low depression levels in the ERP experiment actually were volunteers divided by the cutoff score 50 rather than the selected participants with scores at the two ends of the SDS score. In the revised manuscript with track changes, we have corrected the description in page 8, line 171-176. From the scatter plot with SDS on X axis of Fig 6 in the manuscript, we can also observe that the SDS scores of the two groups were not distributed at the two ends. In addition, we have plotted a unidimensional scatter plot for the SDS scores of all participants, which displayed in SFig 4. It shows the SDS scores are continuous across the two group. Therefore, the SDS scores of all participants can be involved in regression analysis as the dependent variable. In addition, considering the sample size of the high and low depression sub-groups, we have deleted the regression analysis in each group in the revised manuscript and remained the analysis in all participants (page 23, line 480-483).

**SFig 4. The scatter plot for the SDS scores of the two groups.**

**6. The figure was not marked clearly. What’s the figure in three rows represent in Fig4 and Fig5? The statistical value should be written in Fig6.**

**Response:** Thank you very much for the reminder and we are sorry for these omissions. In the Fig 4 and Fig 5, the three rows displayed the waveforms at Fz, Cz and Pz. Followed your suggestion, these marks have been added in Fig 4 and Fig 5. In addition, the R^2^ and the *p*-value in each plot indicating the relationship between the change of LPP and SDS score (or RRS score) in all participants have been added in Fig 6.

**7. In Table3, the early LPP amplitude in the high depression group and the low depression group showed significant differences under the conditions of cp-u and NEU. However, in the middle and late windows, the differences in the high depression group under the conditions of cp-u and NEU gradually disappeared, and the differences in the low depression group varied, which was not explained in the discussion.**

**Response:** Thank you for the constructive advice and we regret for this negligence. The decreased LPP under CP-U condition in the middle and late windows in the high depression group is previously explained as the result of attention distraction (page 26, line 560-561). However, we found that attention distraction might be improper to explain the temporal dynamics of the LPP change under CP-U condition. After literature research, we found that emotion suppression, which is preferred by depressed or depression-prone people in their daily life, might be more appropriate to explain this effect occurred specifically in the middle and late stage (Aldao, et al., 2010). Emotion suppression is a response-focused emotion regulation strategy, which aims to regulate the emotional response after they have been initiated (Liverant, et al., 2008). The temporal property of emotion suppression helps to explain the decreased amplitude in the middle and late windows but not in the early window in the high depression group. The discussion is modified in the page 27-28, line 570-595, and relevant references have also been added in the revised manuscript.

**8. The written English needs considerable improvement.**

**Response:** Thank you very much for the suggestion and we apologize for the problems in writing. We have submitted our manuscript to the *American Journal Experts (AJE)* to modify the errors in writing. We hope the revised manuscript after the re-edition from AJE has been improved in written English.

**Reference:**

Aldao A, Nolen-Hoeksema S, Schweizer S. Emotion-regulation strategies across psychopathology: A meta-analytic review. Clin Psychol Rev. 2010; 30: 217–37.

DeJong H, Fox E, Stein A. Does rumination mediate the relationship between attention control and symptoms of depression? J Behav Ther Exp Psychiatry. 2019; 63: 28–35.

Delorme A, Makeig S. EEGLAB: an open source toolbox for analysis of single-trial EEG dynamics including independent component analysis. J Neurosci Methods. 2004; 134: 9-21.

Derakshan N, Salt M, Koster EHW. Attentional control in dysphoria: an investigation using the antisacccade task. Biol Psychol. 2009; 80: 251–255.

Garnefski N, Kraaij V. Relationships between cognitive emotion regulation strategies and depressive symptoms: A comparative study of five specific samples. Pers Indiv Differ. 2006; 40: 1659–1669.

Gong X, Wang D. Applicability of the international affective picture system in Chinese older adults: a validation study. PsyCh J, 2016; 5: 117–124.

Kaiser J, Schutz-Bosbach. Proactive control without midfrontal control signals? The role of midfrontal oscillations in preparatory conflict adjustments. Biol Psychol. 2019; 148: 107747.

Knyazev GG, Savostyanov AN, Bocharov AV, Rimareva JM. Anxiety, depression, and oscillatory dynamics in a social interation model. Brain Res. 2016; 1644: 62-69.

Koster EHW, Raedt RD, Goeleven E, Franck E, Crombez. Mood-congruent attentional bias in dysphoria: Maintained attention to and impaired disengagement from negative information. Emotion. 2005; 5: 446–455.

Lang PJ, Bradley MM, Cuthbert BN. International affective picture system (IAPS): Affective ratings of pictures and instruction manual. Technical Report A-6. Gainesville, FL: University of Florida. 2005.

Liverant GI, Brown TA, Barlow DH, Roemer L. Emotion regulation in unipolar depression: the effects of acceptance and suppression of subjective emotional experience on the intensity and duration of sadness and negative affect. Behav Res Ther. 2008; 46: 1201–1209.

Palacio-Gonzalez A, Berntsen D, Watson LA. Emotional intensity and emotion regulation in response to autobiographical memories during dysphoria. Cogn Ther Res. 2017; 41: 5305–42.

Semlitsch HV, Anderer P, Schuster P, Presslich O. A solution for reliable and valid reduction of ocular artifacts, applied to the P300 ERP. Psychophysiology. 1986; 23: 695–703.

Yen NS, Chen KH, Liu EH. Emotional modulation of the late positive potential (LPP) generalizes to Chinese individuals. Int J Psychophysiol, 2010;75: 319–325.

Zung WWK. How normal is depression? Psychosomatics. 1972; 13: 174–178.
